# Food poisoning outbreak investigation in Dewachefa woreda, Oromia Zone, Amhara Region, Ethiopia, 2018

**DOI:** 10.1186/s13104-019-4407-9

**Published:** 2019-07-02

**Authors:** Mengistie Kassahun, Sewnet Wongiel

**Affiliations:** 1 Resident of Field Epidemiology, Department of Epidemiology and Biostatistics, College of Medicine and Health Science, Bahir Dar University, Bahir Dar, Ethiopia; 2 Felege Hiote Specialized Referral Hospital, Bahir Dar, Ethiopia

**Keywords:** Food poisoning, Outbreak, Dewachefa woreda

## Abstract

**Objective:**

To verify occurrence of outbreak, describe cases in person, time and place, and identify factors associated with the outbreak. Unmatched case control study was conducted with sample size 175 in Dewachefa woreda from April 24 to May 02/2018. Data were collected with structure questionnaire. Collected data were entered into Epi Info version 7 and exported to statistical package for social science version 23 for analysis. Analyzed data were presented by texts, table and graphs.

**Result:**

A total of 35 food poisoning cases with no death were reported. The overall attack rate was 25.58/10,000. Eating raw meat [adjusted odd ratio (AOR) = 11.04; 95% CI 3.03–40.17], drinking raw milk AOR = 4.81; 95% CI 1.42–16.23), sex (AOR = 3.57; 95% CI 1.37–9.32), hand washing before eating (AOR = 13.42; 95% CI 3.63–49.72) and Sources of drinking water (AOR = 11.50; 95 CI 1.96–67.49) were significantly associated with food poisoning. Food poisoning outbreak were occurred in Dewachfa woreda. Sex of study participants, eating raw meat, drinking raw milk, hand washing before eating, materials use to clean food utensils and source of drinking waters were factors of food poisoning. Proper food handling should be recommended.

## Introduction

Food poisoning is an acute illness with recent consumption of contaminated food or water. It can be infectious or noninfectious. Infectious food poisoning is caused by eating food or water contaminated by bacteria, viruses, parasites or their toxins [1]. It is also called a food born disease [2, 3]. The most common symptoms of food poisoning are nausea, vomiting, abdominal cramps and diarrhea [1–4]. Other symptoms that may occur are fever and abdominal pain [1]. Food poisoning outbreak is the incidence of two or more cases of a similar foodborne disease resulting from the ingestion of a common food [5].

The most common microorganisms that cause food poisoning are Norvirus, Salmonella, *Clostridium perfringens*, Campylobacter and Staphylococcus aurous [1, 5]. Hospitalizations due to food poisoning are mostly caused by Salmonella, Norvirus, Campylobacter, *Toxoplasma gondii* and *Escherichia coli* [1]. Salmonella, *Toxoplasma gondii*, *Listeria monocytogenes*, Norvirus and Campylobacter can cause deaths [1, 2].

Depending on the cause of food poisoning, the duration of the majority of food poisoning usually ranges from a few hours after exposure to contaminated food or fluid to several days [1].

Eggs, poultry, and meats, unpasteurized milk, cheese, raw or unwashed fruits and vegetables, nuts and spices are most commonly associated with food poisoning illness [1, 4]. Factors associated with food poisoning outbreaks also include consumption of inadequately cooked or thawed meat or poultry, cross-contamination of food from infected food handlers, presence of flies, cockroaches, rats, in the food environment that acts as vectors of the disease [6–8].

Globally, 48 million people get sick from a foodborne illness, 128,000 are hospitalized, and 3000 die in each year [9]. Even if the highest number of cases and deaths of food poisoning occur in developing countries [6], there was limited evidence on verification of occurrence the outbreak, the descriptions of the cases and the predictors of food poisoning outbreak in the study area. This investigation aimed to verify occurrence of outbreak, describe cases in person, time and place and identify factors associated with the outbreak in Dewachefa woreda.

On Tuesday morning, April 24/2018, Oromia Zone health department in Amhara Region was contacted by Kemisie General Hospital. In the telephone conversation, Hospital Public Health Emergency Management (PHEM) focal person told Zonal PHEM officer that seven cases of food poisoning had visited Kemisie General Hospital from Qelo and Gure kebeles who were participants of lunch ceremony. Within 30 min of notification of the outbreak by the hospital, four Field Epidemiology Residents, Dewachefa woreda health office and Zonal health department PHEM officers visited the reported hospital and checked the existence of an outbreak. Two Field Epidemiology Resident, woreda health office and Zonal health department PHEM officers visited the village where food poisoning cases outbreak occurred. Other two Field Epidemiology Residents stayed in the hospital to see whether the case is increasing or not.

## Main text

### Methods

#### Study setting and period

Unmatched case control study was conducted from April 24 to May 02/2018 among lunch ceremony participants in Qelo and Gure kebeles, Dewachefa woreda, North-East of Ethiopia. The woreda is located about 325 km from Addis Ababa (the capital city of Ethiopia) and 555 km from Bahir Dar (the capital city of Amhara Region). The total population of the woreda was 151,645. The woreda is administratively divided into 26 kebeles. There are 7 health centers and 26 health post in the woreda. Gure and Qelo kebeles have total population 6001 and 7684 respectively [10]. There was lunch ceremony in Qelo and Gure kebele. The participants were from Qelo and Gure kebeles. About 162 and 50 people were participated in Gure and Qelo kebeles respectively. The outbreak was investigated among lunch ceremony participants in Gure and Qelo.

#### Sample size and sampling techniques

All cases from the two kebeles who participated in lunch ceremony were included in the study with a ratio of one case to four controls. The total sample size was 175 (35 cases and 140 controls). Controls were recruited among participants of lunch ceremony who have not ever an acute illness or sudden onset of abdominal pain, with or without diarrhea, vomiting and nausea. The controls were selected by lottery method simple random sampling in each Kebles.

#### Measurement

Data were collected from both Kemisie General Hospital and house-to-house search for food poisoning case. Structured questionnaires were used to collect data using face to face interview technique by 4 Field Epidemiology Residents. All food poisoning cases that came from Dewachefa woreda to Kemise General Hospital were interviewed. The data were collected using standard questionnaire. Similar codes were written on the cups with questionnaires and the laboratory request formats. Stool samples were collected from 7 food poisoning patients in clean dry cup. These samples were examined using wet mount preparation in Kemise General Hospital. A drop of fresh physiological saline was placed on a clean slide approximately 1 g of stool sample was added. The preparations were covered with cover slips and examined under microscope for the presence or absence of parasites or motile bacteria. In another procedure, a small spot of the stool specimen was placed in a drop of 0.05% methylene blue solution on a clean glass slide for each sample to examine it for cellular exudates.

#### Statistical analysis

Data was entered using EPI-info 7 and analyzed by SPSS version 23. ArcGIS was used for describe the case by place through dots on map. After data cleaning and recoding both descriptive analysis was under taken. Bivariable analysis was done. p-value ≤ 0.25 was included in multivariable analysis. In multivariable analysis p-value < 0.05 was considered significance. Finally results presented in the form of text, tables and figures.

### Result

#### Descriptive epidemiology

##### Distribution by person

A total of 35 food poisoning cases with no death and 140 controls that fulfill standard case definition were included in the investigation. The median age of the cases was 25 + 11.84 standard deviation (SD) year and that of the controls was 28 + 14.54 years. Fifteen (42.9%) of the cases and 95 (67.9%) of controls were 15–44 years old. Three-fifths, 21 (60%), of cases were male whereas 75 (53.6%) of controls were female. All cases have the symptom of vomiting, abdominal cramps and fatigue. Thirty four (97.1%) of cases had history of nausea and diarrhea. The cumulative attack rate was 16.5%. More than half, 20 (57.1%), of cases and three-fourth, 105 (75%), of controls were married.

Regarding to educational status, 16 (45.7%) of cases and 95 (67.9%) of controls had no formal education. More than half, 20 (57.1%) of cases and 80 (57.1%) of controls were farmer (Table 1).

**Table 1:** Characteristics of study participants in Dewachefa woreda, Oromia Zone, Amhara Region, Ethiopia, 2018

Characteristics	Respondents status			
	Cases		Controls	
	Number	Percentage	Number	Percentage
Age in year				
< 5	2	5.7	0	0.0
5–14	6	17.1	30	21.4
15–44	15	42.9	95	67.9
≥ 45	12	34.3	15	10.7
Sex				
Male	21	60.0	65	46.4
Female	14	40.0	75	53.6
Education status				
Not eligible	7	20.0	5	3.6
No formal education	16	45.7	95	67.9
Primary	12	34.3	40	28.6
Occupation				
Not eligible	7	20.0	15	10.7
Farmer	20	57.1	80	57.1
Student	4	11.4	30	21.4
House wife	4	11.4	15	10.7
Marital status				
Not eligible	11	31.4	35	25.0
Single	4	11.4	0	0.0
Married	20	57.1	105	75.0
Eating in other places than usual				
Yes	30	85.7	130	92.9
No	5	14.3	10	7.1
Drink raw milk				
Yes	23	65.7	76	54.3
No	12	34.3	64	45.7
Eat raw meat				
Yes	14	40.0	25	17.9
No	21	60.0	115	82.1
Having latrine				
Yes	30	85.7	110	78.6
No	5	14.3	30	21.4
Using latrine				
Yes	22	73.3	80	64.0
No	8	26.7	45	36.0
Hand washing after defecation				
Yes	5	14.3	100	71.4
No	30	85.7	40	28.6
Hand washing before eating				
Yes	15	42.9	105	75.0
No	20	57.1	35	25.0
Cleaning material for feeding utensils				
Water only	15	42.9	75	53.6
Water with soap	5	14.2	40	28.6
Water with ash	15	42.9	25	17.9
Sources of drinking water				
Pipe	30	85.7	105	75.0
Well	5	14.3	35	25.0
Treat water with chemicals/boil for drinking				
Yes	10	28.6	45	32.1
No	25	71.4	95	67.9
Dispose of garbage				
Pit	10	28.6	55	39.3
Open field	25	71.4	85	60.7

Thirty (85.7%) of cases and 130 (92.9%) of controls were participated in lunch ceremony. Three-fifths, 21 (60.0%), of cases and 115 (82.1%) of controls were eating raw meat. Regarding to milk drinking, 23 (65.7%) of cases and 76 (54.3%) of controls were drinking raw milk (Table 1).

Thirty (85.7%) of cases and 110 (78.6%) of controls had latrine access. Nearly two-thirds, 22 (73.3%), of cases had been using latrine. The Majority of cases, 30 (85.7%) had no hand washing practice after defecation whereas 100 (71.4%) of controls had hand washing after defecation. Furthermore, 20 (57.1%) of cases had no hand washing practice before eating. Twenty five (71.4%) of cases and 85 (60.7%) of controls had practice of disposing house hold solid waste on open field (Table 1).

Regarding to water source, 30 (85.7%) of cases and 105 (75.0%) of controls used pipe water for drinking. More than two-thirds, 25 (71.4%), of cases and 95 (67.9%) controls did not treat water with chemicals/boil for drinking (Table 1).

##### Distribution of cases by place

The outbreak was occurred in Gure and Qelo kebeles, Dewachefa. Regarding to the cases distribution, 28 (80%) of cases were from Gure Kebele. The attack rates of the outbreak in Gure and Qelo Kebeles were 17.3% and 14% respectively.

##### Distribution of cases by time

**Fig. 1:**
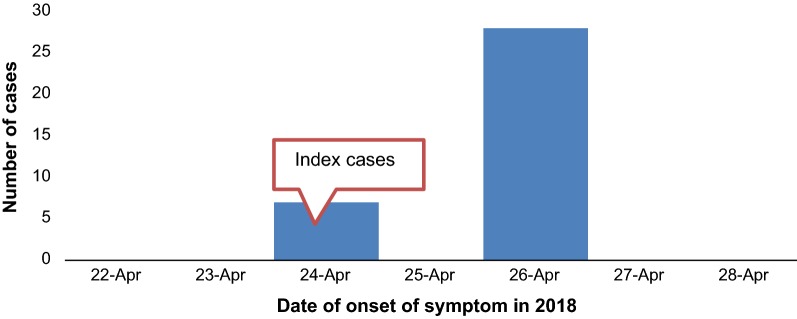
Food poisoning outbreak Epi curve by date of onset in Dewachefa woreda, Oromia Zone, Amhara Region, Ethiopia, 2018

Most cases had onset of symptom on April 26/2018 in which the outbreak was ended (Fig. 1).

#### Laboratory investigation

The results of all wet mount preparation showed that there was no seen parasite or motile bacteria whereas all methylene blue preparation results showed that smaller number pus cells of 20 per high power field were found.

#### Analytical study

In bivariable analysis sex of study participants, eating raw meat and drinking raw milk, hand washing after defecation and before eating, methods/types using to clean food utensils, way of disposing household garbage, source of drinking water use and access of latrine had p-value less than or equal to 0.25 and these variables were included in multivariable analysis.

In multivariable analysis, sex of study participants, eating raw meat, drinking raw milk, hand washing before eating, materials use to clean food utensils and source of drinking waters were significantly associated with food poisoning (Table 2).

**Table 2:** Multivariable analysis of factors associated with food poisoning outbreak in Dewachefa, Oromia Zone, Amhara Region, Ethiopia, 2018

Variables	Respondent status		COR (95% CI)	AOR (95% CI)	p-value
	Case	Control			
Sex					
Female	14	75	1	1	
Male	21	65	2.89 (1.01–12.14)	3.57 (1.37–9.32)	0.009*
Eating raw meat					
No	21	115	1	1	
Yes	14	25	3.07 (2.20–50.41)	11.04 (3.03–40.17)	0.000*
Drinking raw milk					
No	12	64	1	1	
Yes	23	76	1.61 (0.91–10.13)	4.81 (1.42–16.23)	0.011*
Hand washing after defecation					
No	30	40	15.0 (0.65–9.12	5.23 (0.12–10.24)	0.09
Yes	5	100	1	1	
Hand washing before eating					
No	20	35	4.00 (2.75–9.23	13.42 (3.63–49.72)	0.000*
Yes	15	105	1	1	
Cleaning materials for feeding utensils					
Water only	15	75	0.08 (0.02–0.42	0.06 (0.01–0.25)	0.000*
Water with soap	5	40	0.05 (0.01–0.40)	0.06 (0.01–0.32)	0.000*
Ash	15	25	1	1	0.001*
Dispose of garbage					
Pit	10	55	1	1	
Open field	25	85	1.62 (0.07–5.60)	1.01 (0.06–4.54)	0.08
Sources of drinking water					
Pipe	30	105	1	1	
Well	5	35	9.25 (6.01–70.90	11.50 (1.96–67.49)	0.007*

*Statistical significance (p-value < 0.05)

### Discussion

Food poisoning outbreak was occurred among lunch ceremony participants in Dewachefa woreda. This is consistent with the definition of food poisoning outbreak [5]. The etiology of this outbreak was bacteria because the laboratory results showed that pus cells this is one of the indications of bacterial infection. This outbreak specifically might be caused by Salmonella and *Escherichia coli* because of smaller number pus cells of 20 per high power field in the laboratory results.

The highest attack rate was at age of > 44 years. This might be due to the fact that persons whose age > 44 may be influenced by cultures of eating raw meat and drinking raw milk.

The finding of multivariable analysis revealed that eating raw meat, drinking raw milk, not hand washing before meal, materials use to clean food utensils (using soap) and source of drinking water (well water) were independent predictors of food poisoning. People who ate raw meat were almost 11 times more likely had food poisoning illness than people who did not eat raw meat. This is inline with the study conducted in Pima County [8]. This might due to the fact that raw meat has potential to carry foodborne bacteria that can cause illness [1]. The person who prepared the meat might not wash his/her hands which may lead contamination of meat that impose food poisoning illness.

People who drank raw milk were almost 5 times more likely had food poisoning than people who did not drink raw milk. This is similar with the study conducted in western Sweden [8]. This could be due to unpasturazed milk has the potential to carry bacteria which cause food poisoning illness [1, 4]. The milk might be contaminated by bacteria through contamination of the materials in which milk was collected or stored.

This study showed that people who had not hand washing before meal were almost 13 times more likely develop food poisoning illness than who had hand washing before meal. This might be due to contamination of hand with bacteria which leads food poisoning illness through the ingestion of bacteria.

Moreover, those who had been using well water for drinking were almost 11 more likely had food poisoning illness than who had been using pipe water for drinking. This evidence was supported by the study conducted in Pima County [7]. This might be due to the reason that well water would be contaminated with food poisoning agent. Persons who used Water with soap to clean food utensils were almost 94% less likely contracting food poisoning than persons who had used ash for cleaning food utensils. This might due to the fact that soap is the detergent that could clean food poisoning microorganizm.

### Conclusion

Food poisoning outbreak was occurred among lunch ceremony participants in Gure and Qelo kebeles of Dewachfa woreda. The results of this study indicate that sex of study participants, eating raw meat, drinking raw milk, hand washing before eating, materials use to clean food utensils and source of drinking waters were factors of food poisoning outbreak. Therefore, health education should be strength about food handling practice during lunch ceremony.

## Limitation of the study

This outbreak did not show specific etiologic agent because of inavailability other e.g. culture etc.) laboratory services in the hospital. The source of contamination of food was not identified due to inavailability of food sample.

## Data Availability

The data sets generated during the current study are available from corresponding author on reasonable request.

## Abbreviations

AOR: adjusted odds ratio
CI: confidence interval
COR: crude odds ratio
EC: Ethiopian calendar
PHEM: Public Health Emergency Management
SD: standard deviation
SPSS: statistical package for social science
